# Framework for Efficient Auto-Scaling of Virtual Network Functions in a Cloud Environment

**DOI:** 10.3390/s22197597

**Published:** 2022-10-07

**Authors:** Saima Zafar, Usman Ayub, Hend I. Alkhammash, Nasim Ullah

**Affiliations:** 1 Department of Electrical Engineering, National University of Computer and Emerging Sciences, FAST-NU Lahore Campus, Faisal Town B Block, Lahore 54700, Pakistan; 2 Department of Electrical Engineering, College of Engineering, Taif University, P.O. Box 11099, Taif 21944, Saudi Arabia

**Keywords:** auto-scaling, network virtualization, performance of systems, Quality of Service (QoS)

## Abstract

Network Function Virtualization (NFV) offers an alternate method to design, deploy and manage network services. The NFV decouples network functions from the dedicated hardware and moves them to the virtual servers so that they can run in the software. One of the major strengths of the NFV is its ability to dynamically extend or reduce resources allocated to Virtual Network Functions (VNF) as needed and at run-time. There is a need for a comprehensive metering component in the cloud to store and process the metrics/samples for efficient auto-scaling or load-management of the VNF. In this paper, we propose an integrating framework for efficient auto-scaling of VNF using Gnocchi; a time-series database that is integrated within the framework to store, handle and index the time-series data. The objective of this study is to validate the efficacy of employing Gnocchi for auto-scaling of VNF, in terms of aggregated data points, database size, data recovery speed, and memory consumption. The employed methodology is to perform a detailed empirical analysis of the proposed framework by deploying a fully functional cloud to implement NFV architecture using several OpenStack components including Gnocchi. Our results show a significant improvement over the legacy Ceilometer configuration in terms of lower metering storage size, less memory utilization in processing and management of metrics, and reduced time delay in retrieving the monitoring data to evaluate alarms for the auto-scaling of VNF.

## 1. Introduction

Network Function Virtualization (NFV) is a network architecture model that employs virtualization technologies to virtualize the functionality of various network nodes in order to implement communication services. The NFV offers a new way for more open, flexible, and inexpensive networking hardware that is built for general-purpose computing platforms. The industry is intrigued with the possibility of using software-based network functions that are decoupled from hardware through virtualization. Using proprietary hardware makes the service-operator network-vendor-locked as the operators are bound to use products and services from a particular vendor in order to avoid compatibility issues in the network. In the year 2012, a white paper was presented at ‘Software-Defined Networking (SDN) and OpenFlow World Congress’, Darmstadt-Germany through a collective effort of more than twelve companies including AT&T, Verizon, China Mobile, etc. [1]. It was the first time the world had an insight into the NFV; ongoing challenges to the IT industry, potential solutions in the form of NFV, and the challenges faced by the NFV were discussed in detail. In NFV, the dedicated hardware is replaced by general-purpose equipment such as the standard high-volume Ethernet switches, servers, and storage on which virtual appliances run [2]. The network function is implemented in software so that it can run on these general-purpose devices. These devices can be put into network-nodes, data-centers, or at customer premises. Virtual Network Functions (VNFs) are the main components of the NFV framework. These are the building blocks in the NFV architecture. The VNFs are the software implementation of network functions that can be combined together as building blocks to offer a full-scale network communication service. A VNF may consist of one or more Virtual Machines (VMs) running several software and processes, over standard servers, switches, and storage devices including cloud-computing infrastructure [3,4,5]. The NFV paradigm is still in its early stages and there is a large range of possibilities for the research community to design innovative architectures, to develop novel systems and applications, and to examine options and trade-offs in developing technologies for its successful implementation [6].

Cloud-computing or simply ‘Cloud’ is the on-demand services of Information Technology (IT) resources which include computing, storage, and database services. Recent development in cloud-computing technology has given NFV a chance to become a reality. The cloud-computing technologies mainly revolve around virtualization mechanisms, for example, hardware virtualization is conducted through hypervisors that create and run virtual instances. The hypervisor supports several virtual instances on a single host machine by virtually sharing the compute and storage resources among the VMs. Another technology linked with cloud-computing is the use of virtual Ethernet switches; one such example is the virtual switch (vSwitch) which is used to connect traffic between VMs and physical interfaces [7,8,9].

Auto-scaling is a technique used in cloud-computing, whereby the expense of computational resources in a server farm, scales automatically based on the load on the farm. The auto-scaling support has long been added to the traditional cloud [10,11]. Lately, auto-scaling methods have also been proposed and established for the NFV cloud in order to incorporate elasticity in the NFV. The auto-scaling mechanism relates to the management and orchestration of VNFs that can reduce the operational cost for the network service providers. The combination of NFV and cloud-computing technologies makes it possible to perform auto-scaling of VNFs in a cloud environment using cloud management and orchestration. The auto-scaling, i.e., scale-up or scale-down of VNF, works or triggers if some threshold of a VM running in a cloud, is crossed. The threshold value can represent anything related to the VM where the VNF is running, e.g., the Central Processing Unit (CPU) utilization of a VM. The cloud is orchestrated and managed in such a way that it automatically detects these triggers and takes appropriate action. If the load increases on one VM (running VNF), the cloud spins another VM to balance the load. Similarly, if the load decreases to some pre-defined threshold, it automatically turns off the unnecessary VMs, thus efficiently utilizing the available resources [12,13].

There is a need for a comprehensive metering component in the cloud to store and process metrics/samples for efficient auto-scaling of VNFs. The legacy Ceilometer is unable to cope with the amount of data that is stored in its backend storage which results in delays in retrieving the relevant metrics needed to evaluate alarms, thus preventing appropriate action to be executed on time. An effective way to solve this problem is by replacing the Ceilometer metering storage component with Gnocchi, a time-series database. The Gnocchi is designed to solve the storage and indexing problems of time-series data on a large scale. It does this by indexing the resources with proper data types. One of the main features of Gnocchi is to automatically roll up and roll-off measures (time stamp value) based on archive policy due to which the database does not grow out of hand in terms of size. It also performs the aggregation eagerly which results in a quicker response as compared to the legacy Ceilometer which performs aggregation-on-demand resulting in delays [14].

In this paper, we present an integrating framework for efficient implementation of auto-scaling of VNF based on Gnocchi. Our proposed framework reduces the time delay in retrieving metrics from the database that are used in the evaluation of alarms. These alarms are then triggered accordingly to perform auto-scaling of VNF. It is to be noted that Gnocchi is not a replacement for Ceilometer but a metering-storage-part replacement for Ceilometer. To the best of our knowledge, this paper is the first to evaluate the effectiveness of Gnocchi for efficient auto-scaling of VNF.

The main contributions of this paper are summarized as follows:

- We present a comprehensive integrating framework with an efficient metering component in the cloud to store and process metrics for efficient auto-scaling of VNF.
- We examine the proposed framework by implementing VNF in a fully functional cloud by integrating several OpenStack components with Gnocchi, creating Gnocchi archive policies, creating Virtualized Infrastructure Manager (VIM), and defining VNF Descriptor (VNFD).
- By conducting extensive experiments, we verify the efficacy of our proposed framework with respect to the following parameters: (a) predicting the database size of Gnocchi versus Ceilometer for storage of metrics, (b) using timestamp delay, measuring the time it takes to extract monitoring data from the database, (c) comparing memory consumption of Gnocchi versus Ceilometer, and (d) triggering alarms to test the efficacy of our framework for efficient auto-scaling.

The rest of the paper is organized as follows: In Section 2, we discuss the literature review which forms the background for our work. Section 3 presents the detailed design of our proposed framework for the deployment of VNF in a cloud environment. Section 4 presents the results of the empirical analysis of our framework and Section 5 concludes the paper.

## 2. Literature Review

Several research efforts have lately been reported on auto-scaling employing Heat [15,16,17,18,19,20,21]. The details of auto-scaling using Heat are given in [15,16] with policies defined in [17]. In [18], the stack update process is defined, based on which sophisticated automated scaling machines for VNFs can be developed. The document presented in [19] details the Senlin resource types in Heat which make the implementation of a full-featured auto-scaling solution easily achievable. It delivers a tutorial for users for designing a Senlin cluster using Heat. Scaling on OpenStack via Heat with orchestration tools comparison is given in detail in [20]. Recently in the year 2021, Kaur et al., presented a detailed study of OpenStack networking and auto-scaling using a Heat orchestration template [21]. They used OpenStack (Ussuri) released in the year 2020 and demonstrated full-fledged neutron networking along with auto-scaling. They also explained how the Heat orchestration template is employed to define networking infrastructure for enhanced scalability. [22,23,24,25] explain the deployment of auto-scaling using Heat and Ceilometer. In [22], Gupta et al., discuss how OpenStack provides auto-scaling using Heat and Ceilometer. In [23], Yang et al., presented a unique approach to employing Heat and Ceilometers for auto-scaling and we elaborate on it in a subsequent subsection along with its merits and limitations. Gomez-Rodriguez et al., replaced Ceilometer with Monasca which is found to give a superior performance than Ceilometer, on the downside it is difficult to integrate [26]. This auto-scaling is explained in detail in [27]. Recently in the year 2021, Lanciano et al., presented predictive auto-scaling with Monasca and proposed architecture for auto-scaling cloud services based on the status in which the system is expected to evolve in the near future [28]. In addition to SDN-based auto-scaling [29], recently some autonomous VNF auto-scaling methods have been proposed based on artificial intelligence including machine learning and deep learning [30,31,32,33]. In [30], Rahman et al., explore how properties such as the start-up time of underlying virtualization technology affect the Quality of Service (QoS) and cost saving. [32,33] employ deep reinforcement learning and federated learning respectively for auto-scaling.

In the following subsections, we discuss in detail some of the notable approaches for auto-scaling of VNF and also highlight their shortcomings which build the motivation for our work.

### 2.1. Auto-Scaling Using Heat v1.0

**Figure 1:**
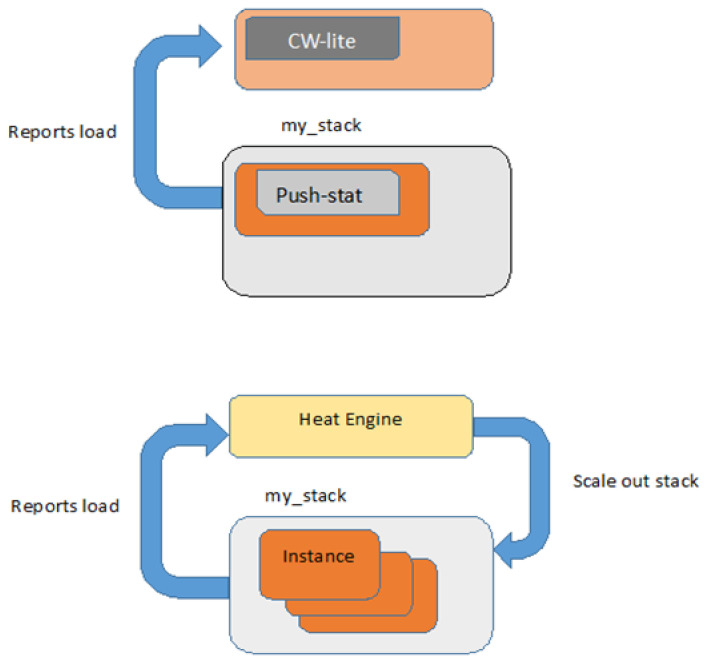
Auto-scaling in Heat 1.0 and Scale-out of VNF.

The OpenStack orchestrates its cloud by using the Heat component [15]. The aim of using the orchestrator is to develop a human-and-machine-accessible service that can be used to control the life-cycle of applications and infrastructure within the OpenStack cloud. The Heat template is used to define the infrastructure of the cloud system. One of the main services provided by the Heat is the auto-scaling service. This service has to be integrated with the telemetry service of the cloud. The researchers of the Heat component at OpenStack put forward a working solution in 2012 to perform auto-scaling of the compute instance by creating and monitoring alarms [16]. The ‘Heat v1.0′ takes a simpler approach for auto-scaling as shown in Figure 1. With the creation of every instance by Heat, a small Python script known as push-stat is also created. The script is scheduled to run for a pre-defined period (with the help of Cron job) and it uses the ‘Util’ package to determine the basic statistics, e.g., CPU utilization or memory usage of the current instances, and report it to the CW-lite. The Heat then runs periodic tasks to determine if the reported metric data of each instance by the Python script have crossed any predefined threshold and if so, the Heat goes either for scale-out as shown in Figure 1 or scale-in of VNF instances based on pre-defined policies.

There are some drawbacks to ‘Heat v1.0′. Although it is capable of implementing auto-scaling, it imposes some undesirable requirements on the Heat component. For instance, it is required that the image that is used to boot up the instances has a small Python script; push-stat available in it which makes images available to Heat to boot up the instances less generic. Another issue with this version is that it uses its local database to store metric data. The metric data has a huge volume and the Heat database is not designed to accommodate such a huge amount of data. There are other issues as well such as the expiration of collected data.

### 2.2. Auto-Scaling Using Heat and Ceilometer

In the year 2016, Yang et al., presented a different approach to implementing auto-scaling [23]. In their proposal, instead of storing data and creating alarms, the Heat instructs the Ceilometer to create and evaluate alarms. Also, the Ceilometer collects data that is relevant to Heat and the data is collected in a convenient way.

**Figure 2:**
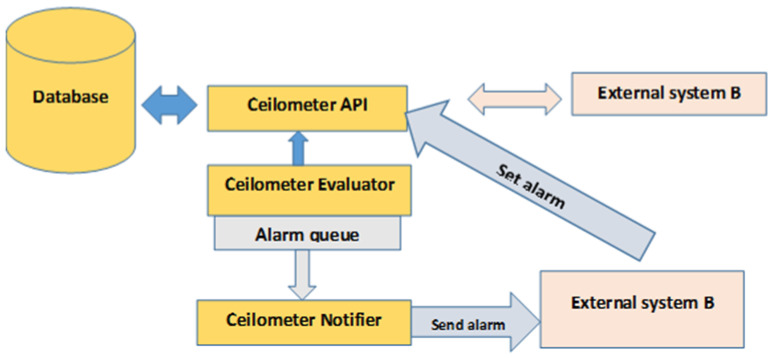
Creation and evaluation of alarms in Ceilometer.

The Heat creates alarms by defining several rules in its template, e.g., the maximum and minimum threshold values on which alarms trigger, the duration window over which the statistics are evaluated, and the comparison operator. The Heat performs these operations via Ceilometer REST API that allows the administrator to control the life cycle of an alarm. Figure 2 shows the creation, evaluation, and notification of alarms. The Ceilometer Evaluator service calls out to the Ceilometer API to obtain alarm definitions that are the rules defined by the Heat template and are stored in the Ceilometer API database. The Ceilometer Evaluator determines whether the threshold has been crossed. If the threshold is crossed, the evaluator uses the Remote Procedure Call (RPC) Notifier API to trigger alarms. The Ceilometer Notifier listens to the RPC bus for alarms alert and executes triggered actions. Here the external system can be Heat that is being informed by the Ceilometer Notifier to perform auto-scaling of VNF by using a pre-defined Uniform Resource Locator (URL) [23,24,25]. One of the main goals of the Ceilometer framework was to store time-series data. In the early development stages of the Ceilometer, it was not clear how the collected data could be manipulated, handled, and queried. As a result, the Ceilometer ended up with a very flexible data model. Although, this helped Ceilometer to become a powerful and handy tool, its performance deteriorated to an extent that it is difficult to store a large amount of data for several weeks without breaking the backend database. The Ceilometer drawbacks can be listed as follows:

1. Large storage footprint
2. Data intake optimization
3. Query API performance issues

The Heat is heavily dependent on the Ceilometer for the creation of alarms and ultimately for auto-scaling of VNF instances. The above-mentioned issues are a source of concern, especially in the production environment. For example, if Ceilometer Evaluator sends a command to the Ceilometer API to obtain the alarm definition provided by Heat or asks the API to retrieve data from the database and if a large amount of data is stored in the database then there is a high probability that the evaluator has to wait for a long time before it obtains a response from the API and sometimes the API request times-out resulting in a failed operation. In the production environment, if Ceilometer is used for data collection and the administrator is unable to retrieve data on time and is unable to react timely on the trigger situations then there is no use for Ceilometer.

### 2.3. Auto-Scaling Using Heat and Monasca

In 2017, Gomez-Rodriguez et al., compared Monasca with Ceilometer by performing different tests [26]. Monasca stands for monitoring-as-a-service that is designed and implemented as a highly-available and horizontally-scalable service. It is considered high-performance and its core components are Apache kafka which is used as a message queue. The other component is Infra DB which is a time-series database. It supports multi-tenant, i.e., it can store measurements, alarm definitions, and notifications. It also integrates with Heat to perform auto-scaling. The authors evaluate the performance of Ceilometer and Monasca by first evaluating the time it takes to retrieve the monitoring data and then comparing their memory utilizations. Monasca gives better results than Ceilometer by consuming less memory and less time in retrieving the monitoring data. On the downside, since Monasca is more independent from OpenStack than Ceilometer, it is more difficult to integrate it with other OpenStack components as compared to the Ceilometer. Monasca also lacks proper documentation on its integration with other components. Deploying an OpenStack cloud is already a challenging task since it is a combination of multiple components that work together to create a complete operational cloud. Monasca has to improve its integration as well as documentation to either replace or work with Ceilometer.

## 3. Deployment of VNF in a Cloud Environment

Our proposed framework comprises of several OpenStack components which are integrated with Gnocchi. We require a fully functional cloud to deploy NFV architecture in-order to evaluate the performance of our proposed framework. Table 1 shows the basic specifications of our test environment.

### 3.1. Proposed Framework

**Figure 3:**
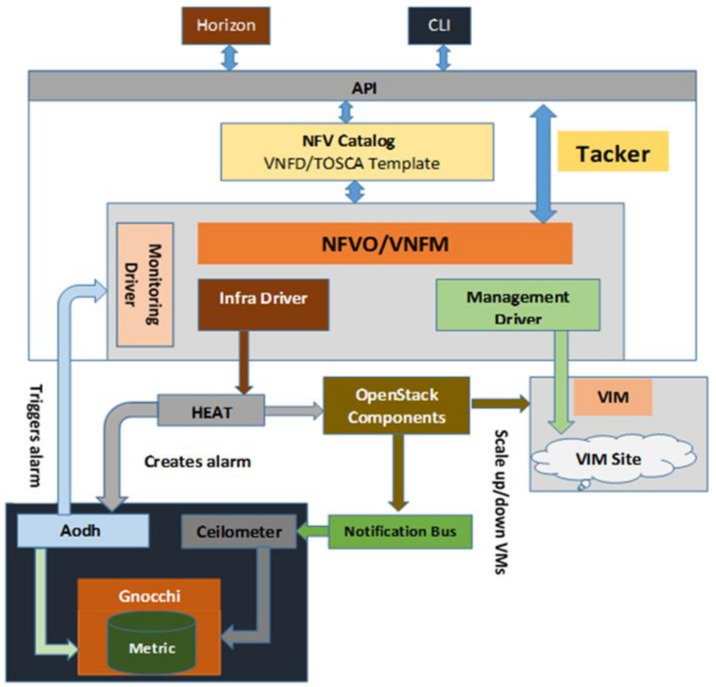
Proposed framework for auto-scaling of VNF.

Figure 3 shows the block diagram of our proposed integrating framework. The cloud environment is implemented in Linux using Ubuntu 16.04.5 Long Term Support (LTS). It is the soundest version of Ubuntu when working with OpenStack components.

**Figure 4:**
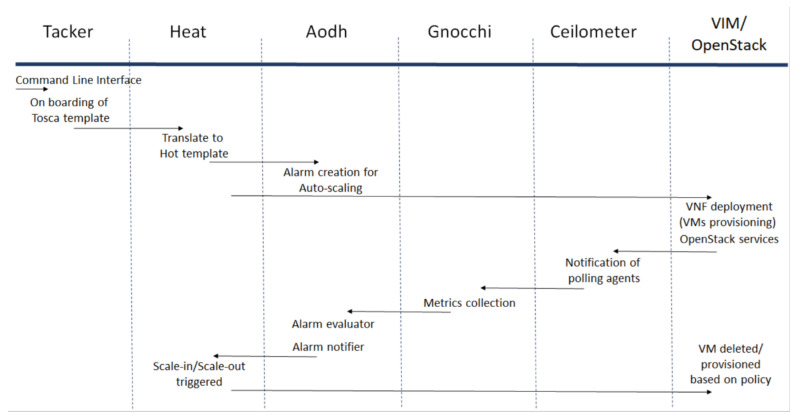
Flowchart of the proposed framework for auto-scaling of VNF.

The OpenStack cloud is deployed using the latest stable version of DevStack (rocky) [34]. The cloud can be controlled by using Command Line Interface (CLI) or Graphical User Interface (GUI). It is always useful to visualize cloud services, operations, etc., and for this purpose ‘Horizon’ framework is selected to provide GUI of the cloud. Tacker is selected as an NFV orchestrator that provides NFV Orchestrator (NFVO) and VNF Manager (VNFM) functionalities to deploy VNF in the cloud. Tacker is selected because most of the components selected in this work are from OpenStack and since Tacker is a product of OpenStack, it is reasonable to believe that it will smoothly integrate with other OpenStack components. Tosca templates are used to define VNF metadata and are used in this work by Tacker [35,36]. The next step is to install Heat which is responsible for auto-scaling of VNF instances. All the information provided in the Tosca template will be translated for the Hot template as Heat uses the Hot template for its operations. Heat also provides relevant information to Aodh for the creation of alarms. The Ceilometer is used to collect telemetry data from OpenStack services and the data is stored in Gnocchi. The flow of the method is shown in Figure 4.

### 3.2. Gnocchi

**Figure 5:**
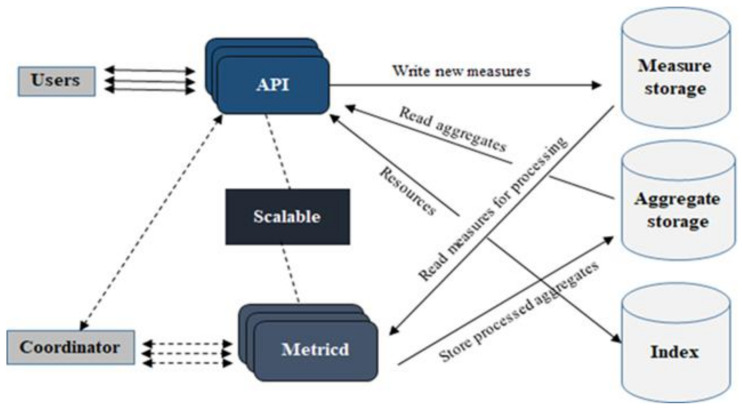
Gnocchi architecture.

Gnocchi has a unique way of storing time-series data. First, it aggregates raw data points and then stores them in the database. Gnocchi archive policies allow us to define aggregation and storage policies for metrics received from the Ceilometer. By default, four archive policies are available. Figure 5 shows an overview of the Gnocchi architecture. There are a number of services; two main services are http server and metric processing daemon or metricd which must be configured before Gnocchi can be used. The http server provides Rest API that is used by the clients to query and write data to Gnocchi. The metricd service is responsible for processing measures sent by the APIs. It also computes aggregates and stores them in aggregate storage. There are three external components which are a part of the Gnocchi architecture and are provided by the drivers; incoming measure storage, aggregated metric storage, and index. Gnocchi gives several options for the storage of incoming measures and aggregated metrics, such as Amazon S3, File (default), Ceph, Redis, and OpenStack Swift. These storage choices can be selected based on the requirement. For medium to large-scale deployment, Redis is recommended for incoming measures and Ceph is recommended for aggregated storage. Swift drivers, S3, and Ceph have additional scalable storage choices. The index stores indices of metrics, resources, and archive policies. It provides storage to their definitions, types, and properties and also links resources with metrics. PostgreSQL and MYSQL are the currently available drivers for index [14].

### 3.3. Creating Gnocchi Archive Policies

The performance of Gnocchi is evaluated against three polling intervals; 60 s 30 s and 10 s. In order to collect data at the mentioned polling intervals, Gnocchi archive policy granularity must be set to the same time interval as the polling intervals. By default, Gnocchi provides three archive policies, the medium archive policy provides a granularity of 60 s. For 30 s and 10 s granularity, we need to create two custom policies. For that purpose, we have made changes in the source code and defined two policies namely delay_30 and delay_10 under the ‘DEFAULT_ARCHIVE_POLICIES’ function. Table 2 enlists the policies that we implemented.

### 3.4. Creating Virtualized Infrastructure Manager

**Figure 6:**
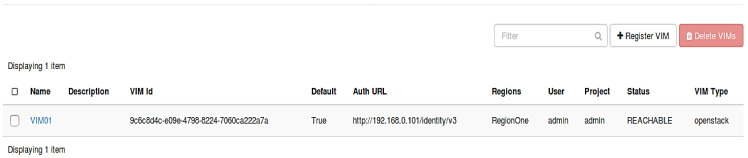
Creation of Virtualized Infrastructure Manager.

Before creating VNF, we need to create VIM. In our proposed framework, VIM is the OpenStack cloud. As discussed earlier, the VIM knows NFV Infrastructure (NFVI) inventory information, e.g., compute, storage, and network capabilities of NFVI. It can directly manipulate NFVI resources by talking to the hypervisor. The VIM can be created by either using OpenStack GUI (Horizon) or by using OpenStack CLI. Figure 6 shows the status of our created VIM. ‘VIM01′ is the name of the newly defined VIM. The authorization URL is provided by Keystone, thus every request for connecting to VIM goes to the Keystone. The VIM type is defined as ‘OpenStack’ and the status shows that the VIM is active and reachable.

### 3.5. Defining VNF Descriptor

VNFD is a template used by Tacker to define the behavioral and deployment information of a VNF. These templates are written in YAML and follow Tosca standards. Virtual Deployment Unit (VDU) is a core part of VNF that hosts network functions. Tosca templates are used to define several properties of VDU such as the image to be used in VDU, its physical properties, VDU monitoring policies, etc. The auto-scaling policies and their thresholds are also defined here. Table 3 shows some of the important properties of VDU defined in our environment.

### 3.6. Testing VNF Deployment

**Figure 7:**
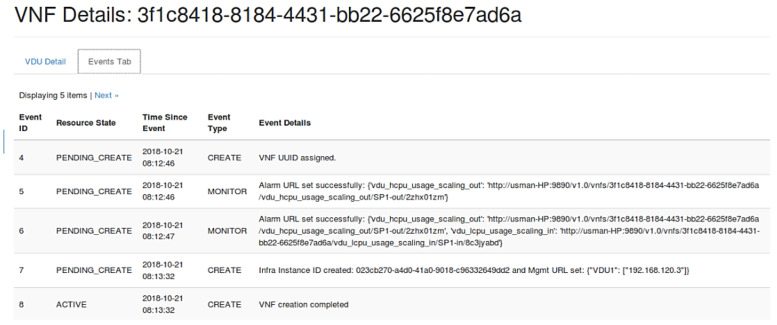
Creation of Virtualized Network Function.

In order to make sure that VNF is properly deployed, we can check VDU details under ‘VNF Manager’ tab in OpenStack GUI (Horizon). Figure 7 shows events and their status while creating VNF and also shows that the VNF is running. Two auto-scaling policies namely ‘vdu_hcpu_usage_scaling_out’ and ‘vdu_lcpu_usage_scaling_in’ are used to create Aodh alarms for auto-scaling of VNF.

### 3.7. Verifying Aodh Alarms Creation

**Figure 8:**
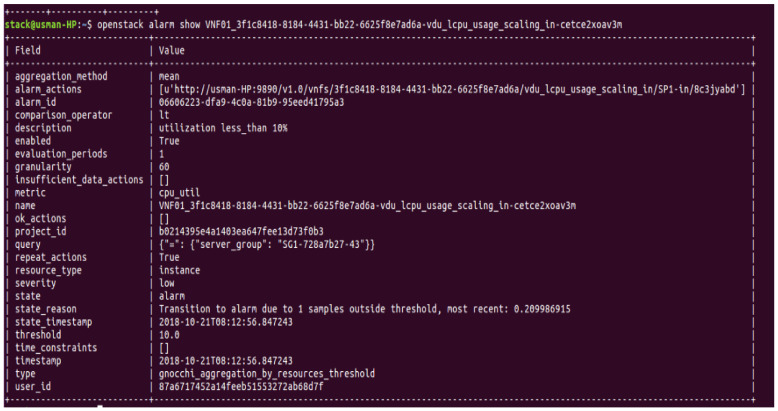
Aodh alarms Scale-in policy parameters.

The monitoring policy described in Tosca templates gives information on creating two alarms for load balancing of VNF. This information is passed to the Aodh component in OpenStack to create and evaluate alarms. To check, if alarms are created successfully, OpenStack CLI is used to display Aodh alarms. The displayed results confirm the creation of two alarms, each for scale-in and scale-out of VNF. The severity option shows ‘low’ status which means none of the alarms are triggered yet. The alarm type is described as ‘gnocchi_aggregation_by_resource_thershold’, i.e., the threshold of alarms is calculated by aggregation methods provided by Gnocchi. OpenStack provides further commands to look into the properties of created alarms. One such command is displayed in Figure 8 with its resulting output. The output shows the properties of the alarm responsible to detect a low threshold which is set to ’10.0′. Another important field ‘aggregation_method’ uses a ‘mean’ function that is applied to all collected points and a single value is compared to set a threshold (10.0) by using the ‘lt’ (less than) operator to check if the threshold is crossed. In case the threshold is crossed, the ‘alarm_actions’ field points to the http link which is used to inform the relevant components (Tacker or Heat) to execute the scale-in policy.

### 3.8. Resulting Network Topology

**Figure 9:**
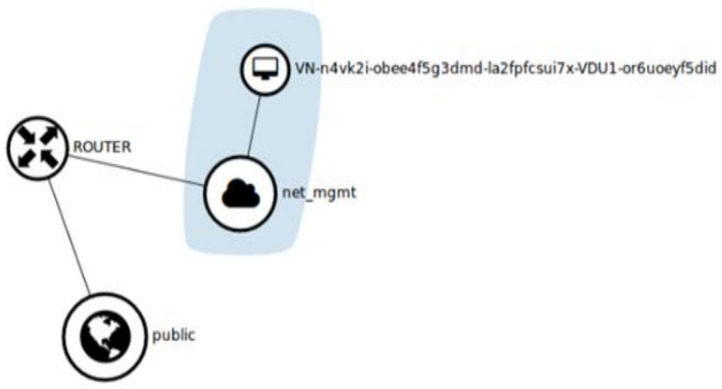
Network Topology after deployment of VNF in a cloud environment.

After completing all necessary deployment, it is time to check the resulting network configuration by accessing the ‘Network Topology’ tab in Horizon. Figure 9 shows the network topology deployment in our cloud. The blue region shows two components; one of which represents the newly created VNF and the other (net_mgmt) is the project network (a virtual private network). The router is used to connect VNF to the public network by assigning floating IPs to it.

## 4. Performance Evaluations

This section explains the results of the performance evaluation of our framework comprising Gnocchi and Ceilometer.

### 4.1. Aggregated Data Points

Aggregated data points are generated from several samples/measures (incoming points that are sent to Gnocchi by the API) according to the defined archive policy. The data points are used to predict the size of the database required by Gnocchi to store metrics. Each point consists of a timestamp and a value. Two case scenarios are used to predict theoretical database size (per aggregate); best and worst. In the best-case scenario, every point occupies a space of 0.05 bytes while in the worst case, the size increases to 8.04 bytes per point. Considering a period of one year, Equation (1) is used to calculate the total number of points collected in a year:

(1) Number of points=timespan/granularity

where timespan = 1 year and granularity = 1 min. The number of points grows in a month. At the end of the first month, the database is populated with 44,640 points that are then increased uniformly each month and reach a maximum value of 525,600 by the end of the year.

### 4.2. Database Size per Metric

After calculating the total number of aggregated points, the next step is to calculate the storage size (in bytes) required to store these points in the Gnocchi database using Equation (2).

(2) Size in bytes=number of points∗ 8 bytes∗ 6

**Figure 10:**
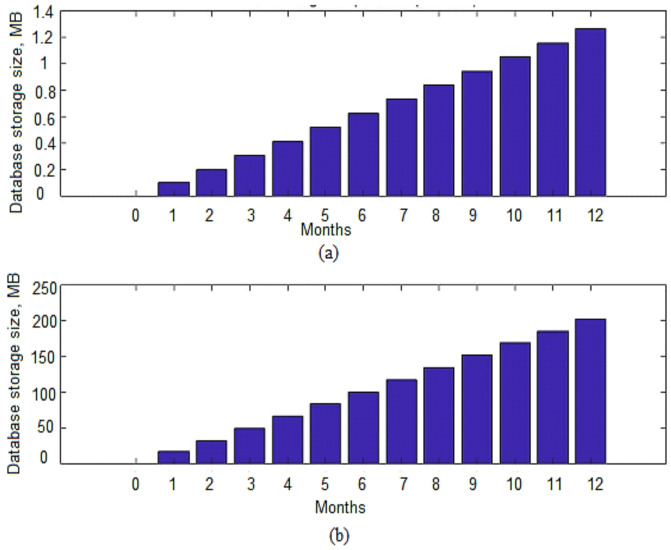
Data storage per metric (**a**) Best-case (**b**) Worst-case.

By default, Gnocchi provides six standard aggregation methods (mean, min, max, sum, std, count). If the defined archive policy uses all of these default methods, the storage space would grow six times as per Equation (2). Figure 10a shows the best-case scenario where the stored data is compressed up to 0.05 bytes per point. At the end of the first month a total of 44,640 points are generated which results in a memory size of 0.11 MB. Similarly, at the end of a year, a total of 525,600 points are generated which take up a space of 1.26 MB. It is to be noted that the storage size is increased by six due to the addition of default Gnocchi aggregated methods. Figure 10b shows the storage size needed for Gnocchi aggregate data points based on the worst-case scenario. The worst-case scenario is more of an interest because, in a production environment, the storage is to be designed in such a way that it can handle the worst imagined situations without creating any interruption to running services. The number of aggregated points increases uniformly with time which results in an increase in memory occupancy. The compressed technique used by Gnocchi is able to store data points into a memory space of 8.04 bytes. At the end of the first month, a total of 44,640 points occupied a space of 17.2 MB while at the end of the year, the data points grow to 525,600 which take up a memory space of 203 MB.

### 4.3. Predicted Database Size of Ceilometer versus Gnocchi

**Figure 11:**
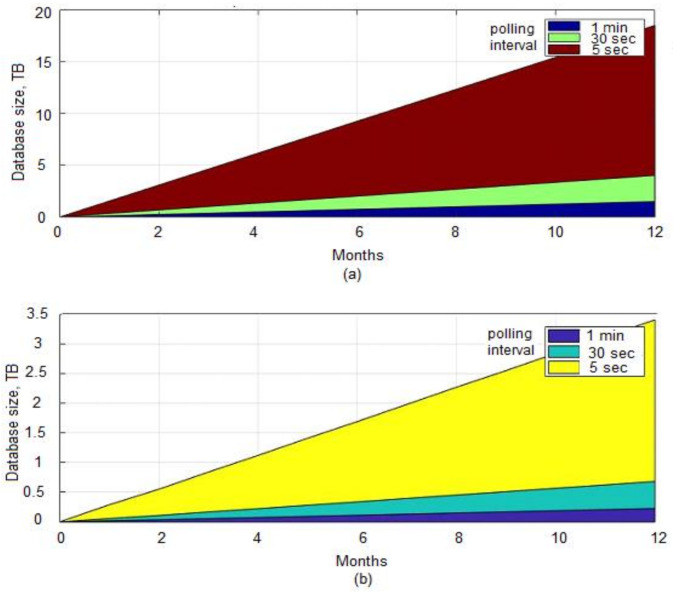
Database size (**a**) Ceilometer (**b**) Gnocchi.

We compare the storage size of the database when using traditional Ceilometer configuration and Mongo DB with a storage size of Gnocchi when using Redis as a storage backend database to save metrics. The Ceilometer database size is predicted using results from [36]. The Gnocchi database size is computed using Equations (1) and (2). Polling is a process of collecting data directly from the OpenStack components APIs, hypervisor, and host machine. Three different polling intervals are used because changing the polling interval causes a significant change in database size. The results are exported to Matlab for visual representation. The parameters are defined as; VMs 360, number of metrics 8, average sample 1.2 Kb, polling interval 60, 30, and 5 s. Figure 11a shows the storage size of Mongo DB when using legacy Ceilometer configuration. With a polling interval of 60 s, after a period of six months, 360 VMs with eight metrics each require a storage size of 0.5 TB while at the end of one year the storage size grows to approximately 1 TB. If polling time is reduced to 30 s, for the first six months the storage size is doubled as compared to the size when the polling interval is 60 s and at the end of a year, more than 2 TB space is needed. Similarly, if we decrease the polling interval further to 5 s, the database shoots up to a size of 7 TB for the first six months and is 14.3 TB at the end of one year. The graph exposes issues with the traditional Ceilometer usage, i.e., the over-growing size of the database which makes it difficult to handle and query the database. As part of our proposed solution, the same parameters are used to estimate the theoretical size of the Gnocchi database, except the backend storage database is changed to Redis.

Figure 11b shows the storage size of the Gnocchi database over a period of one year. A significant decrease in database size can be seen when Gnocchi is employed. For an interval of 60 s, only 0.23 TB size is needed to store a year of data as compared to the 1 TB size required for Ceilometer. Similarly, for a polling interval of 30 s, 0.45 TB size of database is needed as compared to the 2 TB size required for the same period of time when using Ceilometer. Finally, for the polling interval of 5 s, Gnocchi can adjust a year of data in 2.72 TB of space as compared to the Ceilometer storing method which requires a space of 14.3 TB. The major difference in database size is due to the fact that Ceilometer legacy storage stores a full resolution of data and one data point consists of metric and measurement meta-data, time-stamp, IDs, etc. In the case of Gnocchi, only two values are stored per point, i.e., time-stamp and measurement.

### 4.4. Timestamp Delay

**Figure 12:**
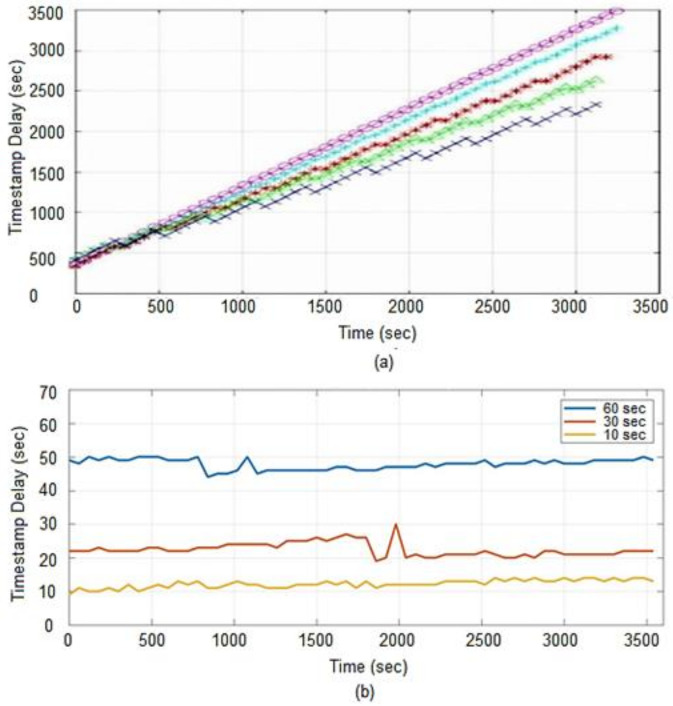
Timestamp delay (**a**) Ceilometer (**b**) Gnocchi.

Timestamp delay is a measure of the time it takes to extract monitoring data from the database. The longer it takes to retrieve metrics, the longer it takes to evaluate alarms and take appropriate action if alarms are triggered. The ideal scenario is to fetch data as soon as it is generated (almost in real-time). The delay is calculated by determining the controller node’s Universal Time Coordinated (UTC) and the last timestamp generated by a metric. In our case, the metric represents the CPU utilization of the compute instance. A small bash script is used to redirect these values to a text file from where the delay is calculated from the given data. The delay is calculated by subtracting controller node time from the latest timestamp of a metric stored in the database. The experiment is conducted on Gnocchi for an hour and the relevant data is directed to the log file from where it is exported to Matlab for graphical representation. A total of sixty-two metrics information is extracted at polling intervals of 60 s, 30 s, and 10 s. In the case of Ceilometer the time stamp delay results are taken from [37]. In this section, this method is applied to one processing/collector worker. These workers are responsible to process incoming metrics to Gnocchi and Ceilometer API. Figure 12a shows that as the polling interval is reduced, the timestamp delay also decreases. The Gnocchi processes the generated metrics very fast, which is why lower polling intervals do not create any backlogs, rather showing low delays compared to large polling intervals. These results are compared with the legacy Ceilometer configuration as shown in Figure 12b. We observe that with polling interval set to 60 s, the timestamp delay in Ceilometer increases to approx. 49 min after an hour as compared to 50 s in Gnocchi. 

In order to determine the effect of the polling period on Ceilometer performance, several polling intervals are used and the result clearly shows that when the polling interval in decreased, the delay in retrieving the data increases. The small polling intervals create backlogs which result in larger delays. In the case of Gnocchi, an opposite behavior is observed as it can retrieve data quicker with smaller polling intervals, e.g., the maximum delay Gnocchi registered when polling every 10 s is 14 s as compared to the Ceilometer delay of 49 min. This is due to the fact that Ceilometer stores everything it can obtain, and in a very inefficient way, therefore, the delay increases significantly with time while the delay in Gnocchi in an hour is insignificant as compared to Ceilometer. The quicker response of Gnocchi is due to the fact that it performs aggregation eagerly which results in a quicker response as compared to the classic Ceilometer that performs aggregation on-demand from a large pile of data stored in a database.

### 4.5. Timestamp Delay with Four Processing Workers

**Figure 13:**
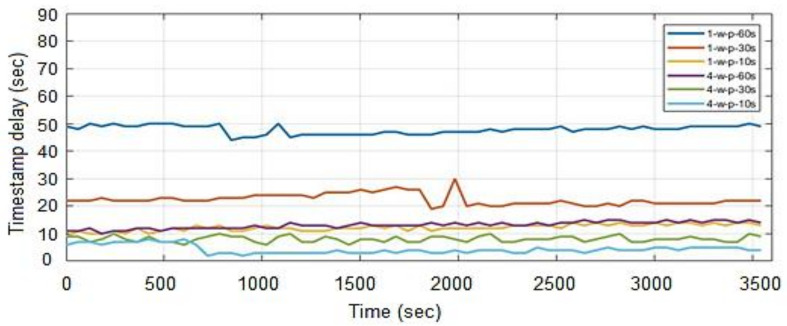
Timestamp delay in Gnocchi with one and four processing workers.

In order to observe the effect of the number of processing workers on timestamp delay when using Gnocchi, another test is conducted by increasing the number of processing workers to four, and results are compared with the output when using one processing worker. The polling is repeated for 60 s, 30 s, and 10 s respectively for a period of one hour. Figure 13 shows a comparison of results when four workers are used as compared to one worker. The results confirm that by increasing the number of processing workers, we can retrieve monitoring data more quickly. The increase in a number of processing workers comes with a price since most of the memory utilization in Gnocchi is due to its processing workers, thus it is interesting to see how increasing the number of processing workers affects the memory of a node.

### 4.6. Memory Consumption

**Figure 14:**
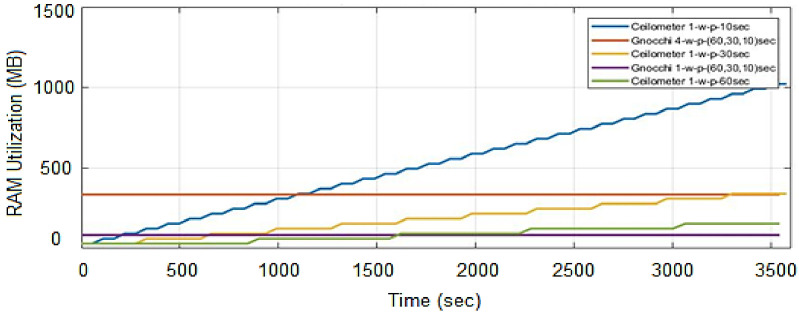
Memory usage comparison of Ceilometer and Gnocchi.

It is observed that the Gnocchi processing worker uses a constant memory of 84 MB for all polling intervals throughout the period while in Ceilometer, the memory usage increases with time in all three polling intervals and is maximum at 1023 MB at a polling interval of 10 s. Thus, in the case of Ceilometer, the memory usage increases when smaller polling intervals are used. In the second configuration, Gnocchi workers are increased to four and it is tested for the three polling intervals. It is observed that the memory usage increases to 336 MB for all polling intervals. Thus Gnocchi utilizes less memory as compared to Ceilometer with the passage of time. Ceilometer, due to its slow operations, result in backlogs which lead to memory issues. In the case of Gnocchi, processing can be scaled horizontally for faster operations by increasing the processing workers. The results are presented in Figure 14. By default, Gnocchi can use all available memory for metric processing but it can be restricted by an administrator. Precautions should be taken when decreasing the available memory to Gnocchi as it can increase the delay in processing metrics that can lead to backlogs.

### 4.7. Triggering Auto-Scaling of VNF

In the previous sub-sections, we compared Gnocchi and Ceilometer and ascertained that Gnocchi is more productive as a metering tool. In this sub-section, we test our proposed framework by trigging alarms to assess the efficiency of auto-scaling in a cloud environment when Gnocchi is used. The auto-scaling of VNF consists of two operations; scale-out and scale-in. The scale-out of VNF is performed in those situations where more VNFs are required to balance out the load on the current VNF. The number of VNFs added when this operation is triggered depends on the policy defined in Tosca templates. The scale-in of VNF is performed when the monitoring policy finds unnecessary VNFs running on nodes. Depending on a predefined policy, VNFs are shut down and resources can be used where needed.

In order to perform auto-scaling, alarms need to be created. These alarms are created using the Aodh component of OpenStack. Two threshold values, 10 and 60 are passed in the Tosca template when creating alarms for scale-in and scale-out operations respectively. In this particular scenario, threshold values represent CPU utilization of VM where VNF is running. The next step is to create a metric name ‘CPU_util’ which is used to store CPU usage for every 60 s. This metric is processed and handled by Gnocchi. Aodh takes this metric from Gnocchi and checks the value to see if the threshold is crossed. In case the threshold is crossed, the alarm triggers, and further information is sent to the Heat component to perform auto-scaling. A Linux command is used to increase the CPU usage of VNF and the data is exported to Grafana to track the usage of the CPU. Horizon (GUI) of OpenStack is used to see if new VNFs are created. Similarly, the command is terminated to see if the scale-in operation is triggered to stop unwanted VNFs.

#### 4.7.1. Scale-Out of VNF

**Figure 15:**
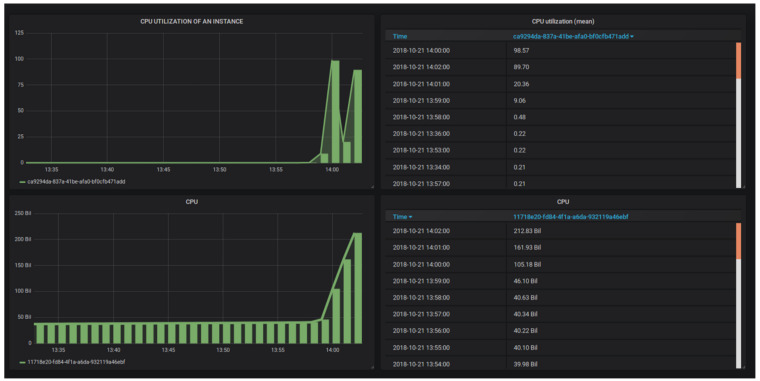
CPU utilization of VNF, Scale-out, and Scale-in.

**Figure 16:**
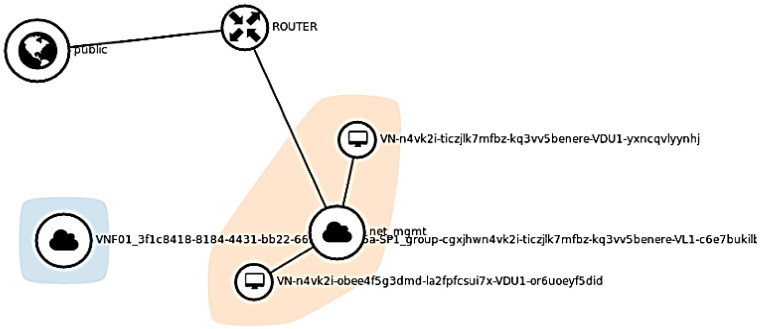
Network Topology for VNF Scale-out.

In order to trigger a scale-out process of VNF, we need to increase CPU utilization of the VM where VNF is running. As per Tosca templates, scale-out initiates if CPU utilization exceeds 60% CPU power. The following command is used in VM to increase CPU utilization: $ dd if=/dev/zero of=/dev/null. The metric (CPU_Util) values are exported in Grafana for a visual representation of CPU consumption by VNF as shown in Figure 15. Figure 15 shows that the CPU usage has reached 98.57%. At this point, an alarm is supposed to be triggered and instructions are expected to be sent to the Heat component to initiate the auto-scaling process. These processes can be tracked by looking at VDU details under the VNF manager tab which shows that scale-out action is initiated by Heat and the ‘Active’ status confirms that the process is completed successfully. According to the defined policy in the Tosca template, the scale-out results in the creation of a new instance/VM where VNF resides. To verify new instance creation, we look in the ‘Instance’ tab and the result shows newly created and default instances. The newly created instance is identified by looking at the ‘time since created’ field which has a value ‘0 min’. In order to confirm, we check the resulting network configuration by accessing the ‘Network Topology’ tab in Horizon which shows the network topology after the addition of a new instance as shown in Figure 16. This includes three components, one of which represents the newly created VNF, the second represents the default instance and the third component (net_mgmt) is the project network (a virtual private network). The router connects VNF to the public network by assigning floating IPs to them.

#### 4.7.2. Scale-in of VNF

In order to trigger the scale-in process of VNF, we need to decrease CPU utilization of the VM where VNF is running. As per Tosca templates, scale-in will initiate if CPU utilization is less than 10%. This is conducted by simply terminating the command (used in the scale-out section), running in VM. As soon as the command is terminated, CPU utilization of VM starts to decrease and reaches 0.20% as evident from Figure 15. In scale-in policy, it is defined to shut down one instance/VM (VNF), since load under 10% can be easily managed by one VNF. In order to observe if the Heat component has triggered the action, we head to the ‘Instance’ tab and its result shows only one instance as the other instance is deleted as a result of the scale-in policy. The resources used by the deleted VNF are put back into their respective pools and are available if needed again.

## 5. Conclusions and Future Work

In this paper, we proposed the use of Gnocchi as a metering storage for performing auto-scaling of VNF in a cloud environment. In order to ascertain the efficiency of our proposed framework, we deployed VNF in a cloud environment and performed extensive tests to establish the efficacy of Gnocchi as compared to the legacy Ceilometer. In order to predict the size of the database, we calculated the total number of aggregated data points, and then computed the storage size required to store these data points in the Gnocchi database. Our results show that Gnocchi outperforms Ceilometer even in the worst-case scenario. The timestamp delay is an important performance parameter that measures the time it takes to extract the monitoring data from the database. Gnocchi gives superior performance in terms of timestamp delay due to faster aggregation. In terms of memory consumption, both for the cases of single and multiple processing workers, it is observed that Gnocchi utilizes less memory than Ceilometer as time passes. We then tested the efficiency of auto-scaling by triggering alarms during scale-out and scale-in of VNF. Our results show a significant improvement when Gnocchi is used. We observe fewer delays when retrieving monitoring data required for evaluating alarms for auto-scaling of VNF. Memory utilization and data storage do not grow out of hand with the passage of time which was the case in the legacy Ceilometer. Based on our results and findings, we claim that Gnocchi is ready to replace the Ceilometer metering storage part in the near future. Through this work, we established the expediency of Gnocchi; a time-series database for contemporary cloud platforms that are large, dynamic, and multi-tenant. The usefulness of Gnocchi for handling large amounts of aggregates being deposited while being scalable and fault-tolerant is also ascertained. We observed that the reason why Gnocchi is fast at recovering data is its feature of storing aggregates instead of raw data points. In the future, we plan to perform more tests on Gnocchi to study memory consumption by Gnocchi workers when the number of metrics/samples is increased. Migration management is also an interesting area of research that involves live VM migration in SDN-enabled Clouds with respect to computing, networking, QoS, and traffic management. Lately, artificial intelligence techniques employing machine learning and deep learning algorithms are being explored for improving efficiency and enhancing the QoS in cloud computing. In the future, we aim to explore machine learning-based techniques for auto-scaling.

## Figures and Tables

**Table 1:** Implementation specifications.

Entity	Condition	Version
Physical server	Processor: IntelI CITM) i5-230M CPU@2.60 GHz RAM: 12 GB Disk space: 256 GB	
OpenStack	Stable	Rocky
Tracker	Stable	Rocky
Operating system	OS Type: 64 bit	Ubuntu 16.

**Table 2:** Archive policies.

Policy Name	Granularity (Gnocchi)	Polling Interval (Ceilometer)
Medium	60 s	60 s
Delay_30	30 s	30 s
Delay_10	10 s	10 s

**Table 3:** VDU Parameters and Policies.

Parameter/Policy	Definition	Value
disk_size	Disk size of virtual machine	1 GB
mem_size	RAM dedicated to VM	512 MB
num_cpus_	Number of CPUs per VM	1
Image	Linux image to boot VM	cirros-0.4.0-x86_64-disk
Metric	store VM CPU utilization values	cpu_util
threshold:	Alarm thresholds	10, 60
aggregation_method	Function applied on collected points	mean
comparison_operator	Greater than, less than, operators	gt, lt
min_instances	Minimum VM to spin	1
max_instances	Maximum VM to be used	2

## Data Availability

Not applicable.

## Abbreviations

API	Application Programming Interface
CLI	Command Line Interface
CPU	Central Processing Unit
GUI	Graphic User Interface
IT	Information Technology
NFV	Network Function Virtualization
NFVI	NFV Infrastructure
NFVO	NFV Orchestrator
QoS	Quality of Service
RPC	Remote Procedure Call
SDN	Software Defined Networking
URL	Uniform Resource Locator
UTC	Universal Time Coordinated
VDU	Virtual Deployment Unit
VIM	Virtualized Infrastructure Manager
VM	Virtual Machine
VNF	Virtual Network Function
VNFD	VNF Descriptor
VNFM	VNF Manager
